# Simvastatin‐Mediated Molecular Mechanisms Underlying the Growth Inhibition of Testicular Leydig Tumour Cells

**DOI:** 10.1111/jcmm.70786

**Published:** 2025-08-28

**Authors:** Arianna De Luca, Lucia Zavaglia, Lucia Francesca Vuono, Francesca Giordano, Davide La Padula, Francesca De Amicis, Vincenzo Pezzi, Adele Chimento

**Affiliations:** ^1^ Department of Pharmacy and Health and Nutritional Sciences University of Calabria Rende Cosenza Italy; ^2^ Department of Health Sciences “Magna Graecia” University of Catanzaro Catanzaro Italy

**Keywords:** apoptosis, cell cycle arrest, insulin‐like growth factor 1 receptor, isoprenoids, Leydig cell tumour, simvastatin

## Abstract

Leydig cell tumours (LCTs) are uncommon stromal neoplasms of the testis, accounting for less than 3% of all gonadal cancers. Most of them are benign, but the malignant ones are very aggressive without specific effective treatment. Several studies reported pharmacologic insight into the use of statins as anti‐tumour agents, but their efficacy on LCTs has not been investigated. Previously, we emphasised the central role of insulin‐like growth factor 1 (IGF1)/insulin‐like growth factor 1 receptor (IGF1R) signalling in Leydig cell tumorigenesis; here, we showed that simvastatin reduces cell proliferation, determines cell cycle arrest at the G1 phase, and induces reactive oxygen species (ROS) accumulation and apoptosis in R2C and LC540 rat Leydig tumour cells. Furthermore, it prevents isoprenoid farnesyl pyrophosphate (FPP) formation and decreases IGF1R expression, leading to the breakdown of the IGF1R signalling pathway. Importantly, we observed that simvastatin synergised with cisplatin in reducing tumour cell proliferation. Collectively, these data suggest that simvastatin is a potential anticancer drug capable of counteracting LCT growth, and it could be proposed as an adjuvant for chemotherapy in LCT treatment.

## 1 Introduction

Leydig cell tumours (LCTs) are rare neoplasms of sex cord/gonadal stromal origin that are mainly characterised by interstitial steroid secretion [1, 2]. These tumours account for about 3% of all testicular neoplasms, with two peaks of incidence: one during the prepubertal age between 5 and 10 years, the other in adulthood between 30 and 60 years old [1]. LCT is usually benign, while an estimated 10%–15% metastasizes and are malignant [3]. Mainly, LCT treatment is surgical resection for both benign and malignant pathological forms; the therapeutic options are very limited and make use of radiotherapy and chemotherapy [1, 3]. Unfortunately, malignant LCTs do not have a good prognosis, respond poorly to chemotherapy or radiation, and they are characterised by an average survival of about 2 years [3]. Although the LCT aetiology is still unknown, in vitro and in vivo studies indicate that overexpression of aromatase, the enzyme responsible for oestrogen synthesis, plays a significant role in Leydig cell tumorigenesis [4, 5, 6]. Furthermore, it has also been observed that LCT cell proliferation is supported by an enhanced expression of insulin‐like growth factor 1 (IGF1) signalling pathway components and by the cooperation between estradiol (E2) and IGF1 [5].

It knows that Leydig cells synthesise steroid hormones starting from cholesterol and that excessive cholesterol accumulation can disrupt their function and potentially lead to impaired steroidogenesis [7]. It is also reported that cancer cells display constitutively elevated levels of 3‐hydroxy‐3‐methylglutaryl coenzyme A (HMG‐CoA) reductase (HMG‐CoAR), resulting in increased cholesterol levels compared to normal proliferating cells [8]. This makes tumour cells potentially more sensitive to a possible depletion of lipid than normal cells; this depletion can be made by statins, a class of cholesterol‐lowering drugs showing different pleiotropic effects including clinically relevant antitumour actions [9]. The cholesterol‐lowering action of statins is due to the inhibition of HMG‐CoAR, an enzyme that catalyses the HMG‐CoA reduction to mevalonate [9]. Although statins are drugs used for lipid disorders treatment, growing evidence supports their potential in prevention and in the treatment of a wide range of cancers, such as brain, hepatic, breast, gastric, colorectal, lung, melanoma, prostatic, head and neck and thyroid [10]. Furthermore, several clinical studies confirm their use as promising antitumour drugs, both in mono‐ and combination therapy [10, 11]. Statins pleiotropic actions affect several cancer cellular processes, such as cell cycle, apoptosis, autophagy, migration, invasion and metastasis [9, 10]; in fact, their antiproliferative, proapoptotic, anti‐angiogenic and anti‐invasive effects were confirmed by in vitro, in vivo and clinical trial studies [10]. The mevalonate pathway plays an important role in cancer development modulation because it also controls the biosynthesis of metabolites which are involved in various biological processes [12]; among these, the geranylgeranyl pyrophosphate (GGPP) and farnesyl pyrophosphate (FPP) intermediates are involved in post‐translational modifications of several guanosine triphosphate (GTP)ase including Ras and Rho [13]. Farnesylation and geranylgeranylation are two lipid isoprenyl modifications involving the farnesyl or geranylgeranyl group attachment to a cysteine residue near the C‐terminus; these reactions ensure the proper membrane association of many proteins which are crucial for signalling, especially in tumour progression [13].

Aim of this study is to investigate whether LCT growth is influenced by simvastatin treatment and to clarify the molecular mechanisms underlying its effects in this tumour type.

## 2 Materials and Methods

### 2.1 Reagents

Dulbecco's modified Eagle's medium (DMEM), HAM–F10, fetal bovine serum (FBS), HS (horse serum), l‐glutamine, penicillin/streptomycin (P/S), dimethyl sulfoxide (DMSO), 3‐[4,5‐dimethylthiazol‐2‐yl]‐2,5‐diphenyltetrazoliumbromide (MTT), phosphate‐buffered saline (PBS) orange acridine (AO), ethidium bromide (EtBr), 2‐(4‐amidinophenyl)‐6‐indolecarbamidine dihydrochloride (DAPI), Bradford reagent, paraformaldehyde, simvastatin (simva), *N*‐acetyl‐l‐cysteine (NAC), ascorbic acid (AA), mevalonate (MEV), farnesyl pyrophosphate (FPP), farnesyltransferase inhibitor‐277 (FTI‐277), insulin‐like growth factor 1 (IGF1) and cisplatin were purchased from Sigma (Sigma‐Aldrich SRL, Milan, Italy). Antibodies against phospho protein kinase B (pAKT), phospho extracellular signal regulated kinase 1/2 (pERK1/2), insulin‐like growth factor 1 receptor (IGF1R), β‐actin, horseradish peroxidase (HRP)‐conjugated secondary antibodies and enhanced chemiluminescence (ECL) western blotting detection system were purchased from Santa Cruz (Santa Cruz Biotechnology Inc., Heidelberg, Germany). Annexin V‐fluorescein isothiocyanate (FITC)/propidium iodide (PI) apoptosis detection kit was purchased from Invitrogen (Thermo Fisher Scientific, MA, USA).

### 2.2 Cells Cultures

R2C and LC540 rat Leydig tumour cells were purchased from American Type Culture Collection (ATCC, Manassas, VA, USA). R2C, a rat testis Leydig tumour cell line derived from a 2‐month‐old male WFu rat, was maintained as previously described [5]. Rat LC540 tumour Leydig cells have been derived from the testis of an 886‐day‐old Fischer rat with bilateral testicular tumours producing large amounts of androgen and oestrogen [14]. This cell line was grown in DMEM with phenol red low glucose supplemented with 10% FBS, 1% glutamine and 1% P/S. All cells were maintained at 37°C in a humidified atmosphere of 95% air and 5% CO_2_. Cell monolayers were sub‐cultured into 6‐well plates for protein and RNA extraction and flow cytometric analyses (2 × 10^5^ cells/well for R2C or 1 × 10^5^ cells/well for LC540), into 12 multi‐well plates for colony formation (10 × 10^2^ cells/well for R2C or 5 × 10^2^ cells/well for LC540) and into 48 multi‐well plates for MTT assay (2.5 × 10^4^ cells/well for R2C or 1 × 10^4^ cells/well for LC540).

### 2.3 Cell Viability Assay

Cell viability was measured using the MTT assay as previously described [15]. Briefly, cells were treated with increasing doses of simvastatin (1.25, 2.5, 5, 10, 20 μΜ for 24, 48 and 72 h), FTI‐277 (2.5, 5, 10, 20, 40 μM for 72 h) or cisplatin (1.25, 2.5, 5, 10, 20 μM for 72 h). Where indicated, simvastatin was combined with NAC (10 mM for R2C; 3 mM for LC540), AA (1 mM for R2C; 0.5 mM for LC540), MEV (200 μM), FPP (10 μM), cholesterol (25–50 μM) or cisplatin (1.25, 2.5, 5, 10, 20 μM). After treatments, fresh MTT, resuspended in PBS, was then added to each well (final concentration 0.2 mg/mL). After incubation (1 h for R2C, or 3 h for LC540), cells were lysed with 200 μL of DMSO and optical density was measured at 570 nm using the Synergy H1 Hybrid Reader (Bioteck S.p.A, Milan, Italy).

### 2.4 Colony Formation Assay

Cells were seeded in 12‐well plates and allowed to grow out in the absence or presence of simvastatin at indicated concentrations for a period of 14 (R2C) or 7 (LC540) days. Colonies were stained with 0.05% Comassie Blue in methanol/water/acetic acid (45:45:10, v/v) (Sigma). The number of colonies was then counted and was normalised to untreated controls (basal).

### 2.5 Flow Cytometric Analysis of DNA Content

R2C and LC540 cells were seeded in six multi‐well plates and then treated for 48 h (R2C) or 24 h (LC540) with simvastatin (5 μM). The cells were harvested by trypsinization and resuspended with 0.5 mL of DNA staining solution (0.1 mg/mL PI, 0.1% sodium citrate and 0.1% Triton X‐100, 0.02 mg/mL RNase; Sigma). The DNA content was measured using a CytoFLEX flow cytometer (Beckman Coulter SRL, Milan, Italy). Nuclei (10000 events) were analysed from each sample. The percentage of cells in the G1, S and G2/M phases of the cell cycle were determined by analysis with CytExpert software (Beckman).

### 2.6 Intracellular Reactive Oxygen Species (ROS) Assessment

R2C and LC540 cells were seeded in 6‐well plates and then treated for 48 h (R2C) or 24 h (LC540) with simvastatin (5 μM). After treatment, the intracellular ROS level in each sample was assessed using the 6‐chloromethyl‐2′,7′‐dichlorodihydrofluorescein diacetate acetyl ester (CM‐H2DCFDA) fluorescent dye (Thermo Fisher Scientific), as previously reported [16]. Samples were then analysed using a CytoFLEX flow cytometer; data were analysed by CytExpert software.

### 2.7 Cellular Morphological Assessment

R2C and LC540 cells were seeded in 6‐well plates (2 × 10^5^ cells/well for R2C or 1 × 10^5^ cells/well for LC540) and then untreated (basal) or treated with simvastatin (5 μM) for 24 h. Subsequent to treatment, culture plates were observed using an inverted phase contrast microscope (Olympus CKX53 inverted microscope, Waltham, MA, USA) and images were captured (20× objective; scale bar = 100 μM).

### 2.8 Determination of Nuclear Morphological Changes

R2C and LC540 cells were seeded in 6‐well plates (2 × 10^5^ cells/well for R2C or 1 × 10^5^ cells/well for LC540) and then untreated (basal) or treated with simvastatin (5 μM) for 48 h (R2C) or 24 h (LC540). Cells were washed with PBS and fixed in 4% formaldehyde for 10 min at room temperature. Fixed cells were washed with PBS and incubated with DAPI (0.2 μg/mL) for 10 min protected from light, at 37°C. Cell nuclei were observed using a fluorescence microscope (Leica DM 6000) (20× magnification, scale bar = 100 μM). Images were acquired and processed using LAS‐X software.

### 2.9 AO/EtBr Staining

R2C and LC540 cells were seeded in 6‐well plates (2 × 10^5^ cells/well for R2C or 1 × 10^5^ cells/well for LC540) and then untreated (basal) or treated with simvastatin (5 μM) for 48 h (R2C) or 24 h (LC540). Cells were harvested by trypsin and centrifuged at 2500 rpm for 5 min to obtain a pellet which was resuspended in 50 μL 1× PBS. Therefore, cells were stained with AO:EtBr (5 μg/mL, 1:1) and incubated for 5 min at 37°C in the dark [17]. Thereafter, 25 μL stained cell mixture was placed on microscopic slides, covered with coverslips, and observed under a fluorescence microscope (Leica DM 6000) (20× magnification, scale bar = 100 μM). Images were acquired and processed using LAS‐X software.

### 2.10 Annexin V‐FITC/PI Staining

Cell apoptosis was assessed by flow cytometry using Annexin V‐FITC/PI apoptosis detection Kit (Thermo Fisher Scientific), according to the manufacturer's instructions. Briefly, R2C and LC540 cells were untreated (basal) or treated with simvastatin (5 μM). After 24 and 48 h, cells were trypsinized, washed once with serum‐containing medium, and resuspended in 200 μL of 1× binding buffer. Then, 100 μL of cell solution was taken and incubated with 2 μL of Annexin V‐FITC for 10 min; then another 100 μL of 1× binding buffer and 2 μL of PI were added for an additional 10 min. After incubation at room temperature in the dark, Annexin V‐FITC binding and PI staining were analysed by CytoFLEX flow cytometer, and data were analysed using CytExpert software.

### 2.11 RNA Extraction, Reverse Transcription and Real Time Polymerase Chain Reaction (PCR)

The RNA extraction was performed as previously described [18]. One microgram of total RNA was reverse transcribed in a final volume of 50 μL using the High Capacity cDNA Reverse Transcription Kit (Thermo Fisher Scientific); cDNA was diluted 1:3 in nuclease‐free water, aliquoted and stored at −20°C. The nucleotide sequences of the IGF1R primers are: forward: GCCTGGTAATCATGCTGTATGTCT; reverse: CACCCCGTTGCCCAATC. PCR reactions were performed in the QuantStudio 3 Real Time PCR System (Thermo Fisher Scientific) using 0.3 μmol/L of each primer, in a total volume of 30 μL reaction mixture following the manufacturer's recommendations. PowerUp SYBR Green Master Mix (Thermo Fisher Scientific) with the dissociation protocol was used for gene amplification. Each sample was normalised to its 18S rRNA gene content. The relative gene expression levels were normalised to a calibrator (basal). Results were expressed as n‐fold differences in gene expression relative to 18S and calibrator, calculated using the 2^−ΔΔCt^ method.

### 2.12 Proteins Extraction and Western Blot Analysis

Whole‐cell lysates were prepared in RIPA Lysis buffer containing protease and phosphatase inhibitors [5]. Protein concentration was determined by the Bradford method. Western blot analysis was performed using monoclonal antibodies against equal amounts (50 μg) of proteins [5]. Blots were incubated overnight at 4°C with antibodies against pAKT (1:1000), pERK1/2 (1:1000) and IGF1R (1:500). Membranes were incubated with HRP‐conjugated secondary antibodies, and immunoreactive bands were visualised with the ECL western blotting detection system. To assure equal loading of proteins, membranes were stripped and incubated overnight with β‐actin (1:4000) antibody.

### 2.13 Statistical Analysis

All experiments were performed at least three times and in triplicate. Data were expressed as mean values ± standard deviation (SD); statistical significance between control (basal) and treated samples was analysed using GraphPad Prism 5.0 (GraphPad Software Inc., La Jolla, CA) software. Control and treated groups were compared using the one‐way analysis of variance (ANOVA) with Bonferroni multiple comparison test post hoc testing. When indicated, nonparametric ANOVA (Kruskal–Wallace test) and post hoc Dunn's test were performed. A comparison of individual treatments was also performed, using one‐tailed Student's *t*‐test (unpaired *t*‐test) or the Mann–Whitney test. Significance was defined as *p* < 0.05.

## 3 Results

### 3.1 Simvastatin Exerts Inhibitory Effects on Leydig Tumour Cell Growth Determining Cell Cycle Arrest at G1 Phase

**FIGURE 1:**
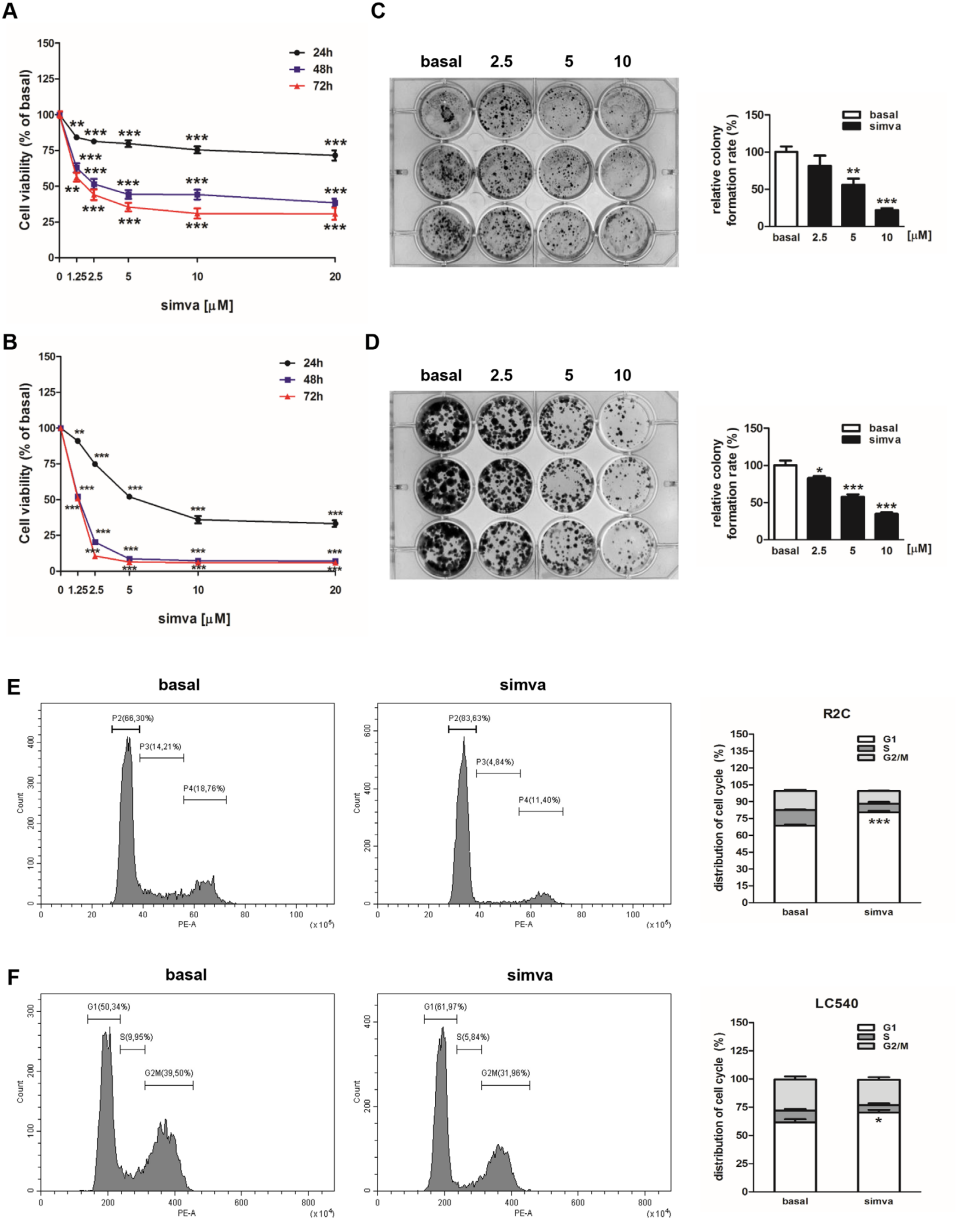
Simvastatin effects on R2C and LC540 cell viability and on cell cycle population distribution. R2C (A) and LC540 (B) cells were treated for 24, 48, and 72 h without (0) or with increasing doses of simvastatin (1.25, 2.5, 5, 10, 20 μΜ) (simva). Cell viability was evaluated by MTT assay as indicated in Section 2. Results are expressed as the mean ± SD of at least three independent experiments (**p < 0.01; ***p < 0.001 vs 0 μΜ). (C, D) Colony formation assay was performed on R2C (C) or LC540 (D) cells plated in the absence (basal) or presence with simvastatin (2.5, 5, 10 μM). Relative colony formation rate (%) was evaluated 14 (for R2C) or 7 (for LC540) days later (*p < 0.05; **p < 0.01; ***p < 0.001 vs basal). Images are from a representative experiment. (E, F) R2C (E) and LC540 (F) were un‐treated (basal) or treated with simvastatin (5 μM) for 24 h (LC540) or 48 h (R2C). Cell cycle distribution was determined by Flow Cytometry using PI stained nuclei. The right graphs show the R2C or LC540 cell population distribution (%) in the various phases of cell cycle. (*p < 0.05; ***p < 0.001 vs basal).

We first investigated whether simvastatin was able to modify the R2C and LC540 cell proliferative behaviour. Our results showed that in R2C cells the use of statin for 24 h determined about a 20% reduction at doses of 1.25, 2.5 and 5 μM and 25% and 30% at the higher doses of 10 and 20 μM, respectively (Figure 1A). In LC540 cells, at the same time, a 10% and 25% inhibition was observed at the lower doses of 1.25 and 2.5 μM, while the higher doses determined a 50% (5 μM) and about 65% (10 and 20 μM) reduction in viability (Figure 1B). In R2C cells, after 48 and 72 h of treatment, the inhibitory effect of the statin was even more evident, causing an inhibition of viability of almost 40% at the 1.25 μM dose (48 and 72 h), of 50% (48 h) and 56% (72 h) at the 2.5 μM dose, and about 60% (48 h) and 70% (72 h) at the 5, 10 and 20 μM doses (Figure 1A). In LC540 cells, simvastatin caused a significant inhibitory effect on cell viability with a reduction after 48 h of approximately 50% (1.25 μM), 80% (2.5 μM) and 90% (5, 10, 20 μM); after 72 h the inhibitory action was equal more or less to 50%, 90% and 95% at the same doses, respectively (Figure 1B). Simvastatin inhibitory effects on Leydig cell growth were also evaluated through the monolayer colony formation assay; we found a significant inhibition starting from the 5 μM dose in the R2C and the 2.5 μM lowest dose in LC540 cells (Figure 1C,D), after 14 (R2C) or 7 (LC540) days of treatment. Cholesterol is very important for cell proliferation and especially for G1/S phase transition [19]. Using flow cytometric analysis we found that simvastatin increased cell population at G1 phase in both R2C (Figure 1E) and LC540 (Figure 1F) cells.

### 3.2 Simvastatin Causes Oxidative Stress in Leydig Tumour Cells

**FIGURE 2:**
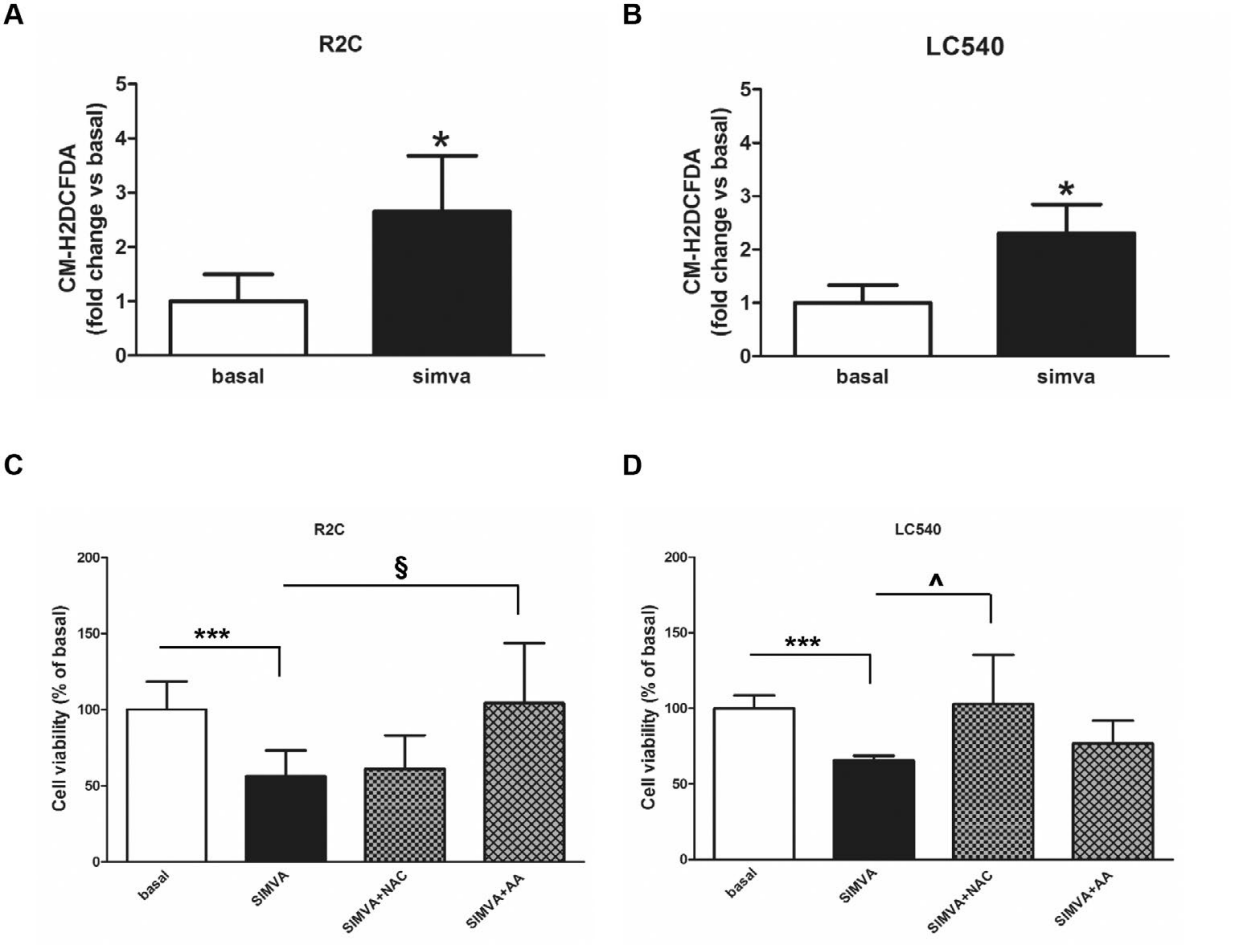
Effects of simvastatin on ROS accumulation in Leydig tumour cells. (A, B) R2C (A) and LC540 (B) cells were un‐treated (basal) or treated with simvastatin (simva) (5 μM) for 24 h (LC540) or 48 h (R2C). ROS levels in R2C and LC540 were quantified by using the CM‐H2DCFDA fluorescent probe. Values represent the mean ± SD of at least three independent experiments (*p < 0.05 vs basal). (C, D) R2C (C) and LC540 (D) cells were treated with 5 μM simvastatin (simva) alone or in combination with AA (1 mM, R2C; 0.5 mM, LC540) or NAC (10 mM, R2C; 3 mM, LC540) for 24 h (LC540) or 48 h (R2C). After treatment, cell viability was assessed by the MTT assay, as described in Section 2. Values represent mean ± SD of three independent experiments (***p < 0.001 vs basal; ^§^ *p* < 0.01, ^^^ *p* < 0.001 vs simva).

Oxidative stress (OS) caused by excessive ROS production has been reported in dying cells [20]. Using the CM‐H2DCFDA fluorescent probe, a significant ROS increase was detected in both R2C (Figure 2A) and LC540 (Figure 2B) simvastatin‐treated cells. Moreover, statin‐dependent cellular inhibition was reverted by the known ROS scavengers AA and NAC, in R2C (Figure 2C) and LC540 cells, respectively (Figure 2D). These results confirm simvastatin‐mediated OS in LCT, also suggesting the ability of the two scavengers to interact with different reactive species as previously confirmed by in vitro assays for both NAC [21] and AA [22].

### 3.3 Simvastatin Triggers Apoptotic Cell Death in Leydig Tumour Cells

**FIGURE 3:**
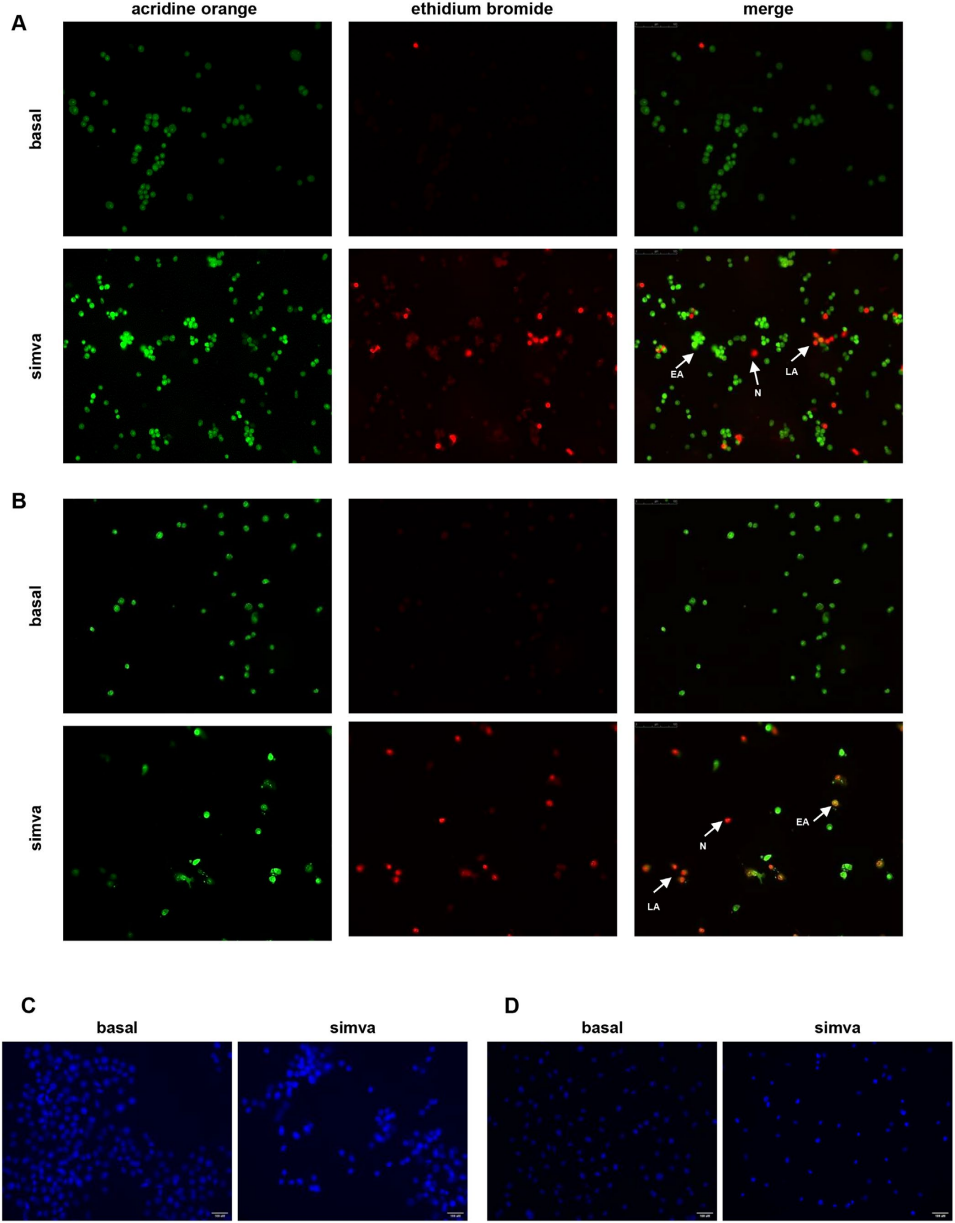
Fluorescence microscopy analysis of cell death in simvastatin‐treated Leydig tumour cells. Cells were un‐treated (basal) or treated with simvastatin (simva) (5 μM) for 24 h (LC540) or 48 h (R2C). (A, B) AO/EtBr staining of R2C (A) and LC540 (B) cells observed under fluorescence microscope was carried out as reported in Section 2. Arrows indicate cells in early apoptosis (EA), late apoptosis (LA) or necrotic cells (N). (C, D) After treatment, R2C (C) and LC540 (D) cells were fixed with paraformaldehyde (4%), stained with DAPI and then examined with a fluorescence microscope (20× objective). Images are from a representative experiment. Scale bar: 100 μm.

**FIGURE 4:**
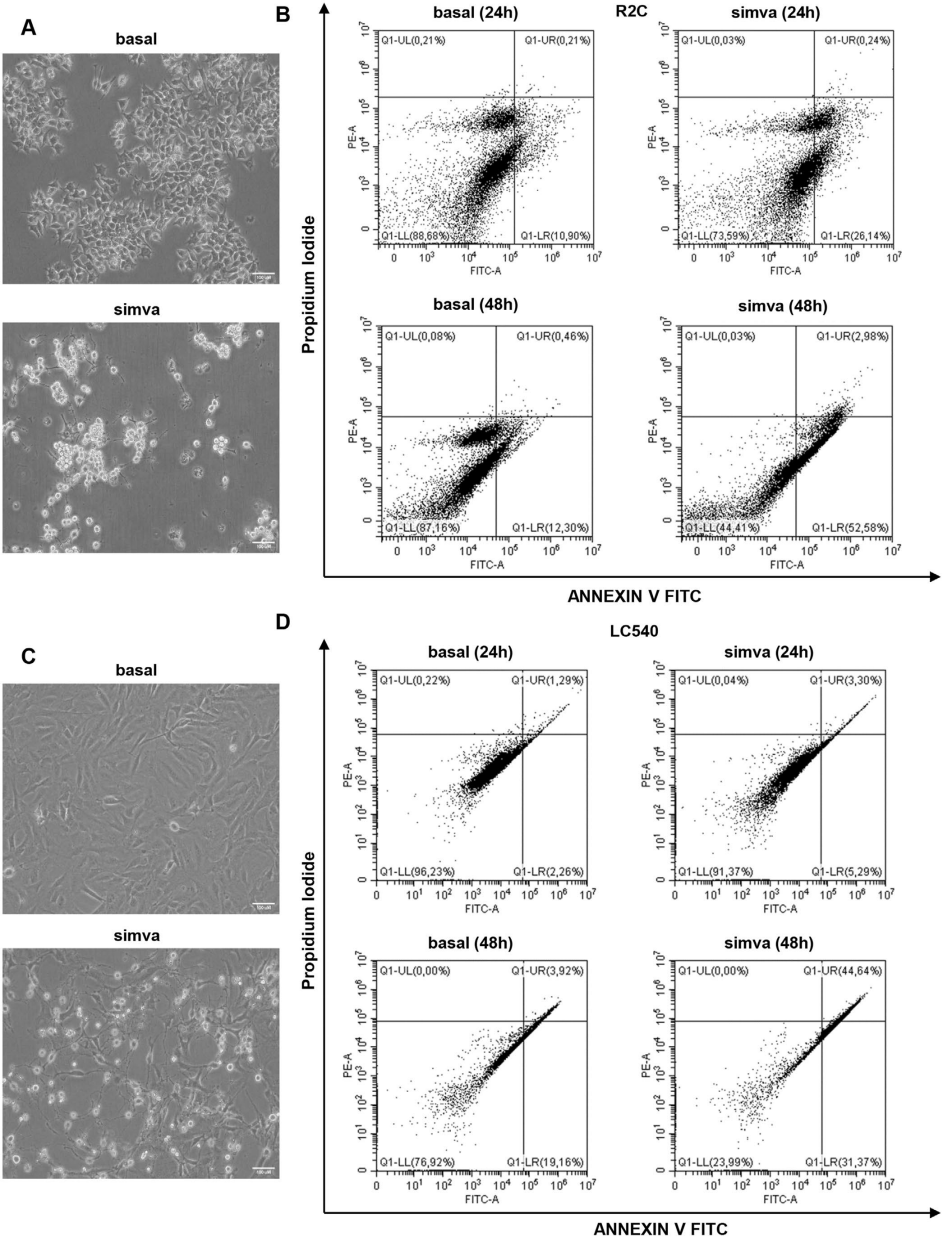
Apoptosis detection in simvastatin‐treated Leydig tumour cells. (A, C) R2C (A) and LC540 (C) cells were untreated (basal) or treated with simvastatin (simva) (5 μM) for 24 h; after treatment, cells were examined with a phase‐contrast microscope (20× objective). Images are from a representative experiment. Scale bar: 100 μm. (B, D) Annexin V‐FITC/PI staining was performed to assess the apoptosis rate in R2C (B) and LC540 (D) cells. After treatment with simvastatin for 24 and 48 h, cells were then stained with Annexin V‐FITC and PI and analysed by flow cytometry. The results are presented as density plots of PI versus Annexin V‐FITC. Apoptotic cells have high Annexin V‐FITC and low PI staining (lower‐right quadrant). Four populations were indicated as viable (lower‐left quadrant), non apoptotic dead (upper‐left quadrant), late apoptotic cells (upper‐right quadrant), and early apoptotic (lower‐right quadrant) cells.

ROS are potential regulators of apoptosis [20]. This cell death type is characterised by both morphological and biochemical changes [23, 24]. AO is a cell membrane permeable dye that penetrates the cell and intercalates into DNA emitting green fluorescence, whereas EtBr enters cells only with cell membrane damage and intercalates DNA emitting red fluorescence. By AO and EtBr staining, live cells show a normal green nuclear fluorescence; early apoptotic cells appear with a bright green nucleus with condensed or fragmented chromatin [25, 26], late apoptotic cells show largely condensed/fragmented orange chromatin, while necrotic cells show deep red nuclei [17, 27]. Our results indicated that simvastatin had the potential to enhance the induction of apoptosis in both R2C (Figure 3A) and LC540 (Figure 3B) cells as evidenced by the presence of bright green/orange chromatin in the cells. Moreover, using DAPI, a blue fluorescent DNA dye, a simvastatin‐dependent strong nuclear condensation was observed in our experimental models (Figure 3C,D). This event was also accompanied by marked changes in both R2C (Figure 4A) and LC540 (Figure 4C) cell morphology; in particular, cellular rounding and loss of cell–cell contacts were evident after statin treatment. Plasma membrane asymmetry loss is an early apoptotic event, characterised by phosphatidylserine (PS) residues exposure at the outer plasma membrane leaflet. Annexin V interacts strongly and specifically with PS and can be used to detect apoptosis [28]. Using Annexin V‐FITC/PI staining, we found that simvastatin increased the apoptotic cell population in a time‐dependent manner. In R2C simvastatin‐treated cells we observed an increase of approximately 2.4‐fold (10.90% in basal; 26.14% in simvastatin treated samples) and 4.3‐fold (12.30% in basal; 52.58% in simvastatin treated samples) after 24 and 48 h, respectively, of the early apoptosis compared to basal (Figure 4B). In LC540, it caused similar effects with a 2.3 and 1.6‐fold enhancement of early apoptosis after 24 h (2.26% in basal; 5.29% in simvastatin‐treated samples) and 48 h (19.16% in basal; 31.37% in simvastatin‐treated samples), respectively (Figure 4D). Moreover, after 48 h of statin exposure, an increase of 6.5 and 13.6‐fold in late apoptosis was obtained in R2C (0.46% in basal; 2.98% in simvastatin‐treated samples) (Figure 4B) and LC540 (3.29% in basal; 44.64% in R2C simvastatin‐treated samples) (Figure 4D) cells, respectively.

### 3.4 Simvastatin‐Mediated Inhibition of Tumour Leydig Cell Proliferation Is Dependent on FPP Depletion

**FIGURE 5:**
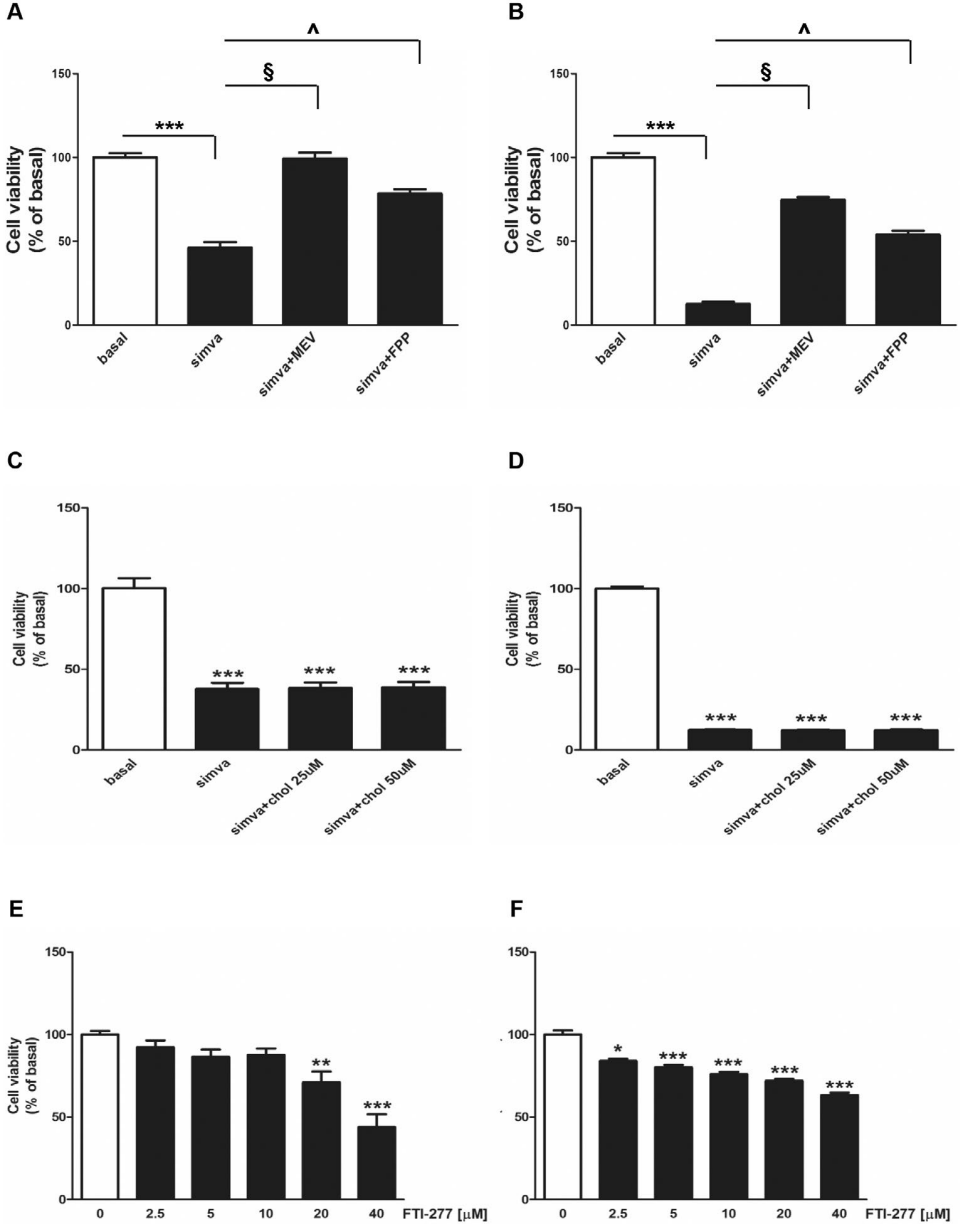
Effects of MEV, FPP, and cholesterol on simvastatin‐mediated cytotoxicity and of FTI‐77 on Leydig tumour cell growth. R2C (A, C, E) and LC540 (B, D, F) cells were untreated (basal, 0) or treated with simvastatin simva (5 μM) for 72 h alone or in combination with (A, B) MEV (200 μM), FPP (10 μΜ) or (C, D) cholesterol (25–50 μΜ), or (E, F) with increasing doses (2.5, 5, 10, 20, 40 μM) of FTI‐277. Cell viability was evaluated by MTT assay as indicated in Section 2. Results are expressed as the mean ± SD of at least three independent experiments. (A–D) ***p < 0.001 vs basal; (A, B) ^§^ *p* < 0.001, simva + MEV vs simva; ^^^ *p* < 0.001 simva + FPP vs simva. (E, F) *p < 0.05; **p < 0.01;***p < 0.001 vs 0 μΜ.

Statin‐dependent cell proliferation inhibition and apoptosis induction can be due to a loss of cell membrane cholesterol and/or depletion of isoprenoid intermediates such as FPP; the latter plays an essential role in anchoring to membranes of small GTPase proteins [13]. In order to verify if simvastatin inhibitory effects were dependent on mevalonate pathway breakdown, the mevalonate and FPP effects were evaluated in both R2C (Figure 5A) and LC540 (Figure 5B) simvastatin‐treated cells. Both compounds were able to significantly reverse the simvastatin‐dependent inhibitory effects on Leydig cell growth (Figure 5A,B). However, the cholesterol use did not have any significant effect on simvastatin‐mediated cell proliferation inhibition (Figure 5C,D). In addition, the effects of FTI‐277, a known farnesyl transferase inhibitor, on Leydig cell viability were evaluated. The observation that this compound decreases R2C (Figure 5E) and LC540 (Figure 5F) cell growth suggested that inhibitory effects of simvastatin in Leydig cells may also depend on FPP depletion.

### 3.5 Simvastatin Inhibits the IGF1/IGF1R‐Dependent Signalling

**FIGURE 6:**
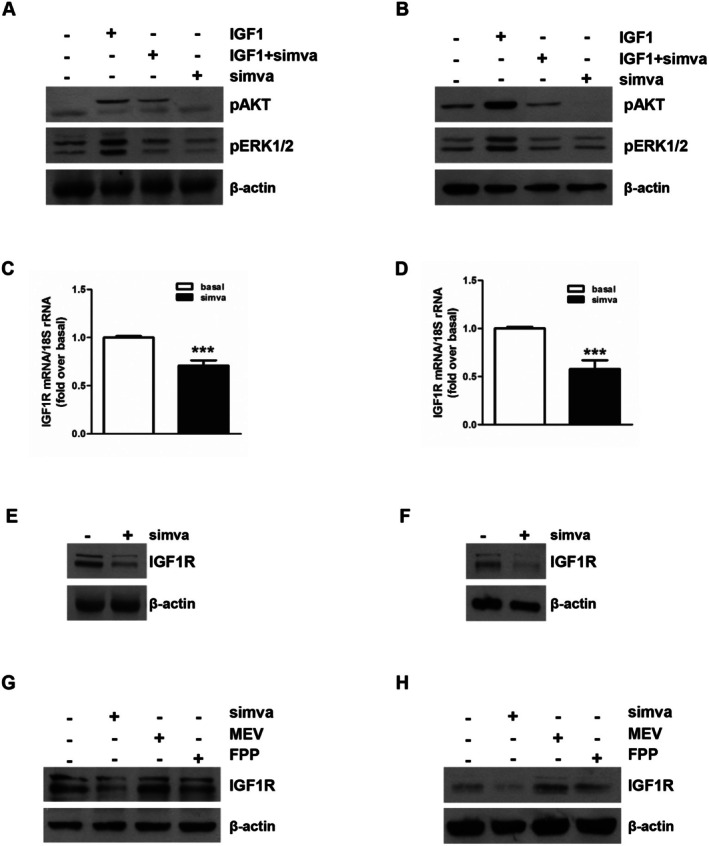
Effects of simvastatin on IGF1R‐mediated signalling pathways and IGF1R expression in Leydig tumour cells. R2C (A) and LC540 (B) were pre‐treated with simvastatin (simva) (5 μΜ) and after exposed to IGF1 (100 ng/mL) for 10 min (for pERK1/2) or 2 h (for pAKT). The AKT and ERK1/2 activation was evaluated by western blot analyses on equal amounts of total proteins. Blots are representative of three independent experiments with similar results. β‐Actin was used as a loading control. (C–F) R2C (C, E) and LC540 (D, F) were untreated (basal, −) or treated with simvastatin (5 μΜ) for 24 h (LC540) or 48 h (R2C). IGF1R mRNA (C, D) and protein expression levels (E, F) were evaluated by real time RT‐PCR and western blot analyses, respectively. (C, D) The IGF1R mRNA expression in every sample was standardised to 18S rRNA content. *N*‐fold differences of gene expression compared to the calibrator (un‐treated sample or basal) were used to graph final results. Data correspond to mean ± SD of values from at least three separate mRNA samples (***p < 0.001 vs calibrator). (E, F) Western blot analysis of IGF1R was performed on equal amounts of total proteins extracted from R2C (E) and LC540 (F). (G, H) R2C (G) and LC540 (H) cells were untreated (−) or treated with simvastatin (5 μΜ) alone or in combination with MEV (200 μΜ) or FPP (10 μΜ).

Small GTPase isoprenylation is required to activate specific receptors and thus kinases signalling cascades involved in cell survival and proliferation [29]. Extracellular signal regulated kinase 1/2 (ERK1/2) and phosphoinositide 3‐kinase (PI3K)/protein kinase B (AKT) can be activated downstream of the IGF1/IGF1R signalling pathway [30]. Our previous data demonstrated that this signalling supports Leydig tumour cell growth [5]. To verify whether the simvastatin‐mediated GTPase isoprenylation inhibition could modulate the IGF1R‐dependent ERK1/2 and AKT activation, we evaluated the effects of simvastatin and IGF1 on the above kinases activation. Simvastatin reduced IGF1‐dependent AKT and ERK1/2 activation in both R2C (Figure 6A) and LC540 (Figure 6B) cells; moreover, a decrease in IGF1R content at both mRNA and protein levels occurs in R2C (Figure 6C,E) and LC540 (Figure 6D,F) cells after simvastatin treatment. Co‐treatment with MEV and FPP reversed these effects in R2C (Figure 6G) and LC540 (Figure 6H) cells, confirming that IGF1R protein expression inhibition was dependent on mevalonate pathway inhibition.

### 3.6 Simvastatin Synergizes With Cisplatin in Inhibiting Leydig Tumour Cell Viability

**FIGURE 7:**
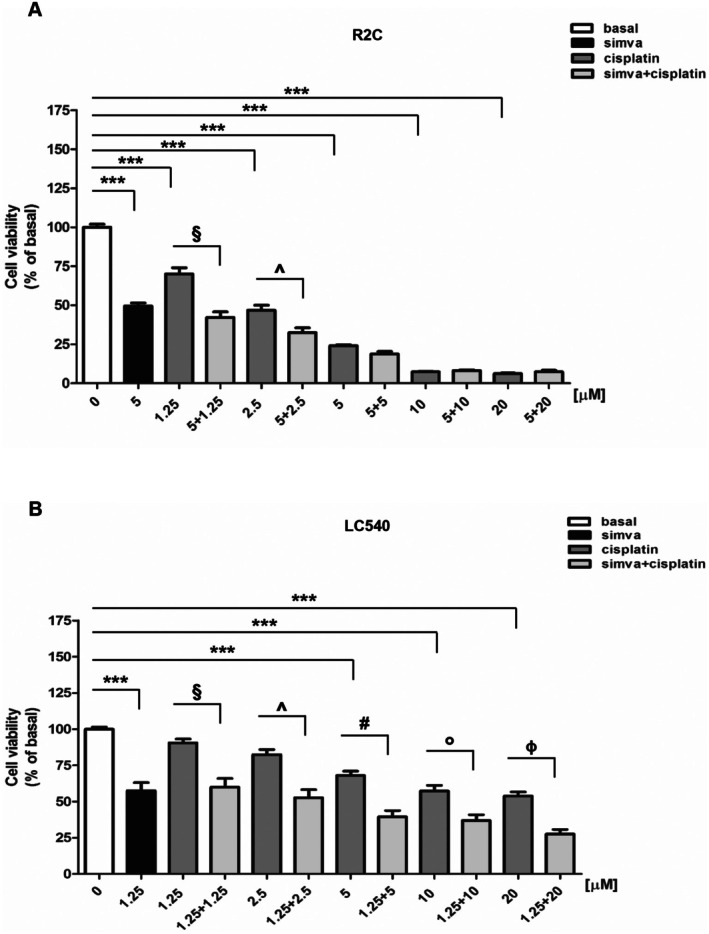
Effects of combined treatment of simvastatin and cisplatin on Leydig tumour cells viability. R2C (A) and LC540 (B) cells were untreated (0) or treated for 72 h with increasing doses of cisplatin (1.25, 2.5, 5, 10, 20 μΜ) or simvastatin (5 μΜ for R2C; 1.25 μΜ for LC540) alone or combined with cisplatin (1.25, 2.5, 5, 10, 20 μΜ). Cell viability was evaluated by MTT assay as indicated in Section 2. Results are expressed as the mean ± SD of three independent experiments (A) ***p < 0.001 vs basal; ^§^ *p* < 0.001 vs cisplatin 1.25 μΜ; ^^^ *p* < 0.01 vs cisplatin 2.5 μΜ. (B) ***p < 0.001 vs basal; ^§^ *p* < 0.001 vs cisplatin 1.25 μΜ; ^^^ *p* < 0.001 vs cisplatin 2.5 μΜ; ^#^ *p* < 0.001 vs cisplatin 5 μΜ; °*p* < 0.05 vs cisplatin 10 μΜ; ^ɸ^ *p* < 0.001 vs cisplatin 20 μΜ.

Finally, we wanted to evaluate the effects of simvastatin and cisplatin combined treatment. In R2C cells (Figure 7A), the 5 μM simvastatin dose was able to determine a greater cell viability suppression when used with the 1.25 and 2.5 μM cisplatin lower doses, compared to cisplatin alone. In LC540 cells (Figure 7B), the combinatory use of 1.25 μM simvastatin with cisplatin was able to significantly potentiate inhibitory effects on cell viability of all doses of cisplatin than when it was administered alone. These results confirmed the synergistic action of both drugs on reducing LCT growth.

## 4 Discussion

In the present study, we demonstrated for the first time that simvastatin‐mediated mevalonate cascade blockade reduced the testicular Leydig tumour cell growth through multiple molecular mechanisms. We observed, in fact, that it suppressed R2C and LC540 Leydig cell proliferation in a dose‐ and time‐dependent manner, and their colony‐forming ability in monolayer. It is known that the cell cycle is often altered in most tumours; therefore, the cell cycle progression regulation is considered an effective approach to control tumour growth [31]. In addition, mevalonate depletion results in cell cycle arrest at the G1 phase and reduction in expression of positive regulators of G1 to S phase progression [32]. Our results from flow cytometric analysis confirmed that simvastatin regulates R2C and LC540 cell cycle distribution, increasing the G1 phase cell percentage. Alterations in intracellular lipid metabolism caused by statins can lead to changes in membrane properties and thus to pro‐apoptotic effects in several cancer types [10]. Furthermore, a variety of anticancer drugs, including statins, can induce apoptosis through ROS generation beyond a critical threshold level leading to OS [33, 34]. Under physiological conditions, ROS production is counterbalanced by their elimination and/or prevention by antioxidant enzymatic systems, ensuring a steady ROS level necessary for the cellular homeostasis maintenance, as it allows them to act as signalling molecules [35, 36]. When this balance is lost, high ROS levels cause oxidative damage to proteins, lipids, DNA, nucleic acids, and other macromolecules, leading to functional disorders and ultimately to cell death [37]. Increased ROS levels can selectively sensitise cancerous and oncogenically transformed cells [20]; in fact, tumour cells have a higher ROS content than normal cells due to their abnormal metabolism, which makes them more susceptible to ROS‐inducing treatment [38]. It has been reported that statin‐induced cytotoxicity in breast [34], cervical [33], colorectal [39] carcinoma is related to OS induction. Statins, by downregulating the antioxidant mevalonate pathway intermediates such as the dolichols, electron transport chain proteins heme A and ubiquinone (coenzyme Q10), cause severe OS [40] that contributes to improving chemotherapy efficacy [41]. Our results demonstrated that simvastatin causes ROS accumulation in both the used experimental models; its cytotoxic effects are reversed by NAC and AA, two known scavengers of reactive species, in both LC540 and R2C cells, respectively, thus confirming simvastatin‐induced OS in Leydig tumour cells. Moreover, the observed nuclear and cellular morphological changes after simvastatin treatment suggested an obvious signal of apoptotic cell death. These events are, in fact, associated with Annexin V‐FITC/PI and AO/EtBr staining positivity confirming apoptosis induction. The simvastatin ability to trigger this cell death event is in agreement with our previous results in the adrenal carcinoma cell line, where the reduction of intratumoral cholesterol content was able to prevent E2 production, inhibit the mitochondrial respiratory chain, and ultimately induce apoptosis [42]. Cancer cell proliferation inhibition and apoptosis induction by statins can be due to a loss of cell membrane cholesterol and/or depletion of isoprenoids [42, 43]. Our results indicated that mevalonate and FPP use are able to rescue statin‐treated cells from cell viability inhibition. However, statin‐induced cytotoxicity is not directly dependent on cholesterol synthesis inhibition, since cholesterol addition was unable to prevent this effect. This agrees with other reports showing that statins inhibit cell growth independently of cholesterol availability [44]. Conversely, mevalonate supplementation in the presence of statins totally restored cell proliferation in R2C and partially in LC540. Since mevalonate is downstream of HMG‐CoA, it is likely that statins effects are due to reduced levels of intermediates in the cholesterol synthesis pathway downstream of mevalonate but upstream of cholesterol, such as FPP, which is responsible for GTPase proteins farnesylation, such as Ras [13]. Ras farnesylation is required for its plasma membrane translocation; only membrane‐associated Ras is able to interact with membrane receptors, resulting in activation of downstream signalling cascades regulating cell growth and survival [29]. Since oncogenic Ras mutations have been found in several human cancers [45], the efficacy of FTase inhibitors in cancer treatment was evaluated [46]. We found that treatment with FTI‐277, a known farnesyl transferase inhibitor, was able to inhibit both Leydig tumour cell lines viability; this observation confirmed that farnesylation is crucial for proliferative events in Leydig tumour cells. Furthermore, Ras farnesylation correlated with membrane receptors activation and then survival kinases signalling cascades induction [29] such as ERK1/2 and PI3K/AKT kinases that can be activated downstream of the IGF1/IGF1R signalling pathway [30].

The IGF1/IGF1R system dysregulation is involved in the proliferation of numerous tumours [47] as well as the IGF1/IGF1R system inhibition by statins, which may be of preventive and/or therapeutic value in some tumours such as prostate [48]. Our previous studies demonstrated that one of the molecular mechanisms determining Leydig cell tumorigenesis is excessive oestrogen production that stimulates a short autocrine loop sustaining cell proliferation; moreover, cell‐produced IGF1 amplifies oestrogen signalling through a steroidogenic factor‐1 (SF‐1)–dependent up‐regulation of aromatase expression [5]. Here, we demonstrated that simvastatin treatment interfered with IGF1R‐mediated signalling by reducing both IGF1R expression and IGF1‐dependent AKT and ERK1/2 activation. Our data agree with other reports demonstrating that simvastatin was able to reduce IGF1R expression in PC‐3 prostate and SK‐MEL‐2 melanoma cell lines [48, 49]. It has been reported that IGF1R expression is closely related to cholesterol metabolism. A possible mechanism for mevalonate‐regulated cell growth is the involvement of dolichol, a product of the mevalonate intermediate FPP; this molecule mediates the transport of proteins (e.g., IGF1R) to the ER lumen for their N‐glycosylation; the latter is an important event for protein correct translocation to the cell membrane [49]. Studies reported that mevalonate and the intermediate products of the cholesterol biosynthesis pathway induce the expression of IGF1R, which can significantly improve the anti‐apoptotic capacity of tumour cells [50]. Therefore, our results indicate simvastatin as a potent inhibitor of the IGF1/IGF1R system and suggest its potential use in the preventive and/or therapeutic treatment of LCT. Preclinical studies showed that the combinations of statins with anticancer drugs or cytotoxic chemotherapy agents exhibit synergistic effects on various cancer cell types [51]. However, the statin‐dependent molecular mechanisms underlying the increased toxicity of antineoplastic agents are not entirely clear. The combinatory use of simvastatin and chemotherapy agents augmented anticancer responses against several cancers [10]. It sensitised murine MLTC1 Leydig tumour cells to etoposide by increasing gap junction intercellular communication (GJIC) composed of connexin 43 (Cx43); this effect is related to the inhibition of protein kinase C (PKC)‐mediated Cx43 phosphorylation at serine 368 and subsequent increase in Cx43 membrane localization [52]. Moreover, clinical trials investigating combination therapy with statins displayed encouraging results with better outcomes compared to monotherapy [11]. Our results showed that simvastatin was able to increase the responsiveness of R2C and LC540 to cisplatin. An explanation of this phenomenon could be due to the ability of simvastatin to increase plasma membrane fluidity and then facilitate the drug's active and/or passive diffusion, as observed in previous studies [53]. Another hypothesis could be that the statin combination would help overcome resistance mechanisms involving variation in drug efflux [54].

In conclusion, taken together, our results suggest that simvastatin could be an effective potential anticancer drug capable of counteracting Leydig tumour cell growth through multiple pleiotropic effects. These include the OS generation and apoptosis induction, depletion of mevalonate pathway intermediates (i.e., FPP) which are involved in the prenylation of proliferative pathways regulatory molecules, and IGF1R‐mediated signalling inhibition. Moreover, the observation that it synergises with cisplatin to reduce Leydig cell viability proposes it as an adjuvant to enhance the action of chemotherapy agents in LCTs.

## Author Contributions

**Arianna De Luca:** data curation (equal), investigation (equal), methodology (equal), validation (equal), writing – original draft (equal). **Lucia Zavaglia:** investigation (equal), methodology (equal), validation (equal). **Lucia Francesca Vuono:** investigation (equal), methodology (equal), validation (equal). **Francesca Giordano:** investigation (equal), methodology (equal), validation (equal). **Davide La Padula:** investigation (equal), methodology (equal), validation (equal). **Francesca De Amicis:** validation (equal), writing – review and editing (equal). **Vincenzo Pezzi:** conceptualization (equal), data curation (equal), funding acquisition (equal), investigation (equal), methodology (equal), supervision (equal), validation (equal), writing – review and editing (equal). **Adele Chimento:** conceptualization (equal), data curation (equal), investigation (equal), methodology (equal), supervision (equal), validation (equal), writing – original draft (equal), writing – review and editing (equal).

## Ethics Statement

The authors have nothing to report.

## Conflicts of Interest

The authors declare no conflicts of interest.

## Data Availability

The data that support the findings of this study are available from the corresponding author upon reasonable request.
